# Clinical Performance of Direct RT-PCR Testing of Raw Saliva for Detection of SARS-CoV-2 in Symptomatic and Asymptomatic Individuals

**DOI:** 10.1128/spectrum.02229-22

**Published:** 2022-11-21

**Authors:** Rosa Castillo-Bravo, Noel Lucca, Linyi Lai, Killian Marlborough, Galina Brychkova, Maryam Shideh Sakhteh, Charlie Lonergan, Justin O’Grady, Nabil-Fareed Alikhan, Alexander J. Trotter, Andrew J. Page, Breda Smyth, Peter C. McKeown, Jelena D. M. Feenstra, Camilla Ulekleiv, Oceane Sorel, Manoj Gandhi, Charles Spillane

**Affiliations:** a Genetics & Biotechnology Lab, Ryan Institute, National University of Ireland Galway, Galway, Republic of Ireland; b Quadram Institute Bioscience, Norwich Research Park, Norwich, Norfolk, United Kingdom; c College of Medicine, Nursing and Health Sciences, National University of Ireland Galway, Ireland; d Health Service Executive (HSE) West, Merlin Park University Hospital, Galway, Ireland; e Thermo Fisher Scientific, South San Francisco, California, USA; Quest Diagnostics

**Keywords:** asymptomatic, COVID-19, d, RT-PCR, SARS-CoV-2, saliva, surveillance, symptomatic

## Abstract

RT-PCR tests based on RNA extraction from nasopharyngeal swabs (NPS) are promoted as the “gold standard” for SARS-CoV-2 detection. However, the use of saliva samples offers noninvasive self-collection more suitable for high-throughput testing. This study evaluated performance of the TaqPath COVID-19 Fast PCR Combo kit 2.0 assay for detection of SARS-CoV-2 in raw saliva relative to a lab-developed direct RT-PCR test (SalivaDirect-based PCR, SDB-PCR) and an RT-PCR test based on RNA extraction from NPS. Saliva and NPS samples were collected from symptomatic and asymptomatic individuals (N = 615). Saliva samples were tested for SARS-CoV-2 using the TaqPath COVID-19 Fast PCR Combo kit 2.0 and the SDB-PCR, while NPS samples were tested by RT-PCR in RNA extracts according to the Irish national testing system. TaqPath COVID-19 Fast PCR Combo kit 2.0 detected SARS-CoV-2 in 52 saliva samples, of which 51 were also positive with the SDB-PCR. Compared to the NPS “gold standard” biospecimen method, 49 samples displayed concordant results, while three samples (35<Ct<37) were positive on raw saliva. Among the negative samples, 10 discordant cases were found with the TaqPath COVID-19 Fast PCR Combo kit 2.0 (PPA–83.05%; NPA–99.44%), compared to the RNA extraction-based NPS method, performing similarly to the SDB-PCR (PPA-84.75%; NPA-99.63%). The direct RT-PCR testing of saliva samples shows high concordance with the NPS extraction-based method for SARS-CoV-2 detection, and therefore provides a cost-effective and highly scalable system for high-throughput COVID-19 rapid-testing.

**IMPORTANCE** The scale of the COVID-19 pandemic highlighted the need for viral diagnostic systems that are accurate and could be deployed at large population scales. Large-scale diagnostic or surveillance testing of large numbers of people requires collection of infected biological samples that is easy and rapid. Here, we demonstrate that raw saliva samples can be easily collected and tested by RT-PCR assays. Indeed, we find that direct testing of raw saliva by two different RT-PCR assays is as accurate (if not more accurate) than nasal swab-based RT-PCR testing. We present a cost-effective and highly scalable system for high-throughput COVID-19 rapid-testing.

## INTRODUCTION

The emergence of severe acute respiratory syndrome coronavirus 2 (SARS-CoV-2) in Wuhan in 2019 led to a global pandemic of coronavirus disease 2019 (COVID-19). SARS-CoV-2 can lead to both symptomatic and asymptomatic infections, making detection of infected individuals challenging if based solely on symptomatic diagnostic testing. To combat viral spread and ensure public health, countries have implemented different strategies related to diagnostic, screening, and surveillance testing. COVID-19 tests should exhibit high sensitivity and quick turnaround times to adapt treatment, reduce the spread of disease, and adjust public health interventions to the local epidemiology. Establishing COVID-19 testing in high-throughput settings such as schools or workplaces also requires tests that are easy to use, require minimal resources, and have a high acceptance rate by the individuals involved in the testing (1).

Detection of SARS-CoV-2 in RNA extracted from nasopharyngeal swab (NPS) samples using quantitative RT-qPCR is considered to be the gold standard for identification of COVID-19 infection, as the virus typically infects the upper respiratory tract. However, reliable collection of NPS requires trained health care professionals, and NPS samples can be difficult to obtain from some individuals due to the discomfort associated with the technique. Using saliva as an alternative sample type to NPS offers several advantages, including noninvasive self-collection, reduced risk of viral transmission, and lower sample costs in terms of trained health care personnel, personal protective equipment, and costs associated with sample collection (2).

A number of studies have shown that saliva and NPS RT-PCR-based tests exhibited comparable analytical performance (3–10). In addition, several reports indicate that saliva might be more sensitive than nasopharyngeal or nasal swabs for diagnosis of SARS-CoV-2 infection, especially for asymptomatic cases or with the emergence of new SARS-CoV-2 variants that can have a different tropism compared to earlier variants (7, 11–13). Indeed, the 2021 guidance on the use of saliva as sample material for COVID-19 testing highlighted the potential of saliva for nucleic acid based (i.e., PCR based) SARS-CoV-2 testing, while cautioning on the use of saliva as a sample for rapid antigen or antibody tests (14).

The aim of this retrospective study was to evaluate the performance of the TaqPath COVID-19 Fast PCR Combo kit 2.0 and our SalivaDirect-based (SDB) RT-PCR protocol in raw saliva specimens in comparison to the NPS RNA extraction-based TaqPath COVID-19 CE-IVD RT-PCR which is considered to be the gold standard for the detection of SARS-CoV-2.

## RESULTS

### RT-qPCR on raw saliva shows concordance with RT-qPCR on NPS-extracted RNA for SARS-CoV-2 screening.

**FIG 1:**
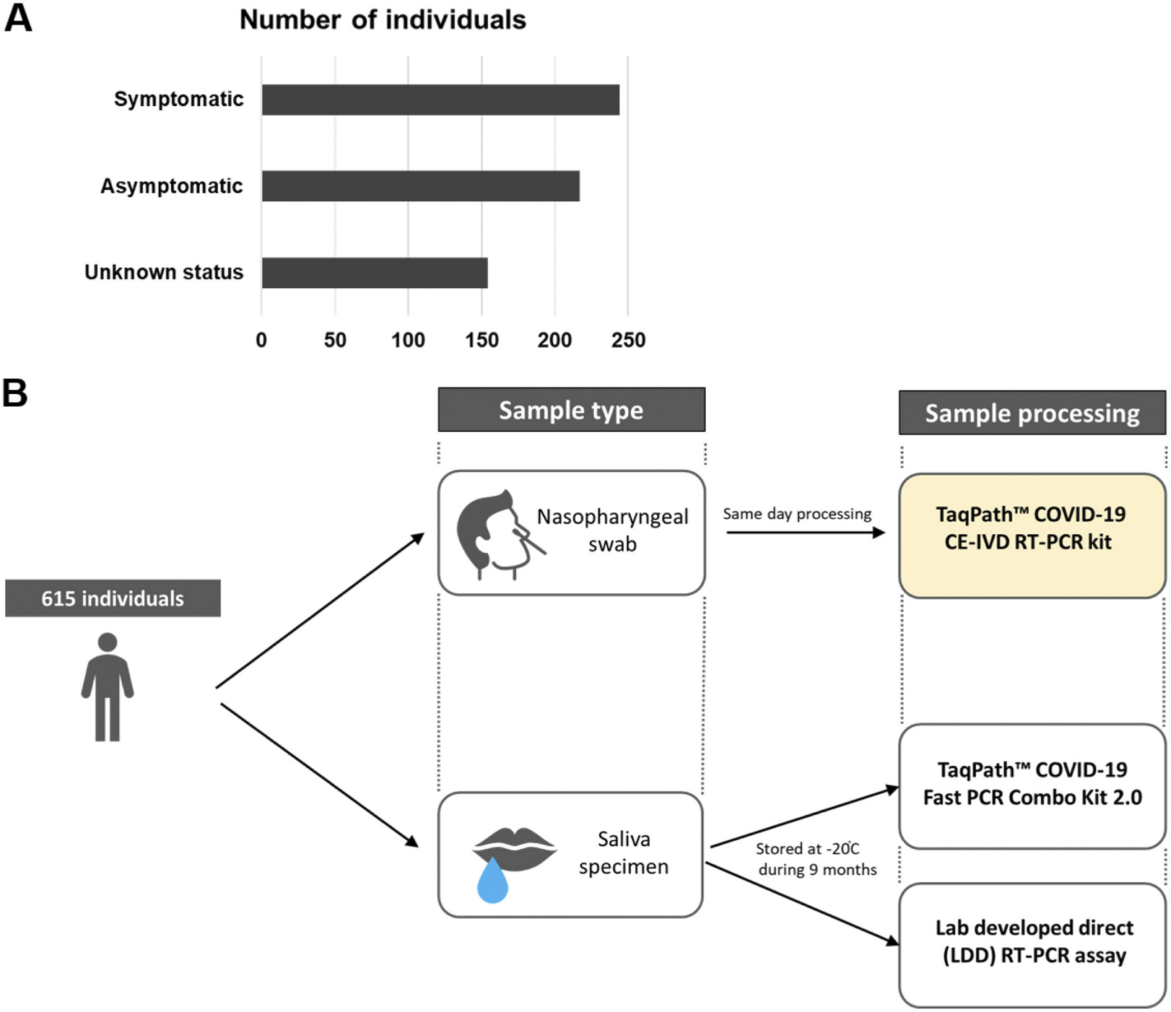
Study design. (A) Cohort description based on the symptomatic status. (B) A total of 615 individuals provided saliva and nasopharyngeal swab samples on the same day. Samples were processed according to the algorithm shown.

A total of 615 raw saliva samples obtained from symptomatic and asymptomatic individuals were tested following long-term storage at −20°C using both the TaqPath COVID-19 Fast PCR Combo kit 2.0 and the lab’s SDB-PCR test. At the time of saliva sample collection, all individuals also provided NPS samples which were tested using an extraction-based RT-qPCR test in an HSE diagnostic laboratory. For all individuals in the study, the result of the RT-qPCR test from the nasopharyngeal swab sample was available. All raw saliva samples were tested both at the time of collection and again following long-term storage at −20°C using the SDB RT-qPCR assay without an RNA extraction step (Fig. 1B). For all samples matching results were obtained at the time of sampling and at the time of repeated testing following long-term storage using the SDB RT-qPCR, indicating that no deterioration of sample had occurred.

To evaluate the performance of the direct RT-qPCR testing approach of raw saliva for detection of SARS-CoV-2, results obtained by testing with the TaqPath COVID-19 Fast PCR Combo kit 2.0 were compared to the results based on the nasopharyngeal swab testing using an extraction-based RT-qPCR method (Table 1).

**TABLE 1:** Positive and negative percent agreement of the raw saliva-based testing using the TaqPath COVID-19 Fast PCR Combo kit 2.0 and the nasopharyngeal swab-based testing using an RNA-extraction RT-PCR diagnostic assay^*a*^

Direct RT-qPCR assay	Saliva result	Extraction based RT-PCR from nasopharyngeal swab samples		
		Positive	Negative	Total
TaqPath COVID-19 Fast PCR Combo kit 2.0	Positive	49	3	52
	Negative	10	534	544
	Total	59	537	596
Positive Percent Agreement (PPA)		83.05% [71.54% to 90.52%]		
Negative Percent Agreement (NPA)		99.44% [98.37% to 99.81%]		

*^a^*Each individual provided one saliva and one nasopharyngeal swab sample on the same day. The nasopharyngeal swabs were processed on the same or following day, while the saliva testing was performed on samples following storage at −20°C for several months.

SARS-CoV-2 was detected using the TaqPath COVID-19 Fast PCR Combo kit 2.0 in 52 raw saliva samples from the cohort panel, of which 51 were in full agreement with both the SDB-PCR results at the time of collection and retesting following storage at −20°C. Interestingly, two samples tested positive only from raw saliva (33 < Ct < 37). In both cases they tested positive consistently for both the TaqPath COVID-19 Fast assay (Ct = 32.9 and 36.85 for N1) and the SDB-PCR (Ct = 33.3 and 36.8 for N1), while the RNA extraction-based testing of the NPS in these cases yielded a negative result.

The performance of the lab’s SDB RT-qPCR in raw saliva samples was also evaluated in comparison to the nasopharyngeal swab testing using an extraction-based RT-qPCR method (Table 2) and performed similarly to the TaqPath COVID-19 Fast assay.

**TABLE 2:** Positive and negative percent agreement of the raw saliva-based testing using the SDB RT-PCR assay and the nasopharyngeal swab-based testing using an RNA-extraction RT-PCR diagnostic assay^*a*^

Direct RT-qPCR assay	Saliva result	Extraction based RT-PCR from nasopharyngeal swab samples		
		Positive	Negative	Total
SDB RT-PCR assay	Positive	50	2	52
	Negative	9	535	544
	Total	59	537	596
Positive Percent Agreement (PPA)		84.75% [73.48% to 91.76%]		
Negative Percent Agreement (NPA)		99.63% [98.65% to 99.90%]		

*^a^*Each individual provided one saliva and one nasopharyngeal swab sample on the same day. The nasopharyngeal swabs were processed on the same or following day, while the saliva testing was performed on samples following storage at −20°C for several months.

### Whole-genome sequencing data show predominance of B.1.1.7 variant lineage.

Whole-genome sequencing data were obtained for 46 of the SARS-CoV-2 positive samples. As expected based on the variants circulating in the Republic of Ireland during the period of sample collection (between February 8 and May 6, 2021), the vast majority of the positive samples consisted of the B.1.1.7 lineage (N = 45), with one sample identified as the B.1.562 lineage. When the SARS-CoV-2 clade was determined, 91.1% of the positive samples belonged to the 20I (Alpha, V1) clade, while 8.9% of the samples consisted of the 20A clade. WGS data were available for one of the two samples that showed positivity using both saliva-based testing methods (36 < Ct < 37 for N1) while negative on RNA from NPS and identified the presence of the Alpha VOC in the sample.

### Saliva-based testing offers good performance for different SARS-CoV-2 detection, including at low viral burden.

**FIG 2:**
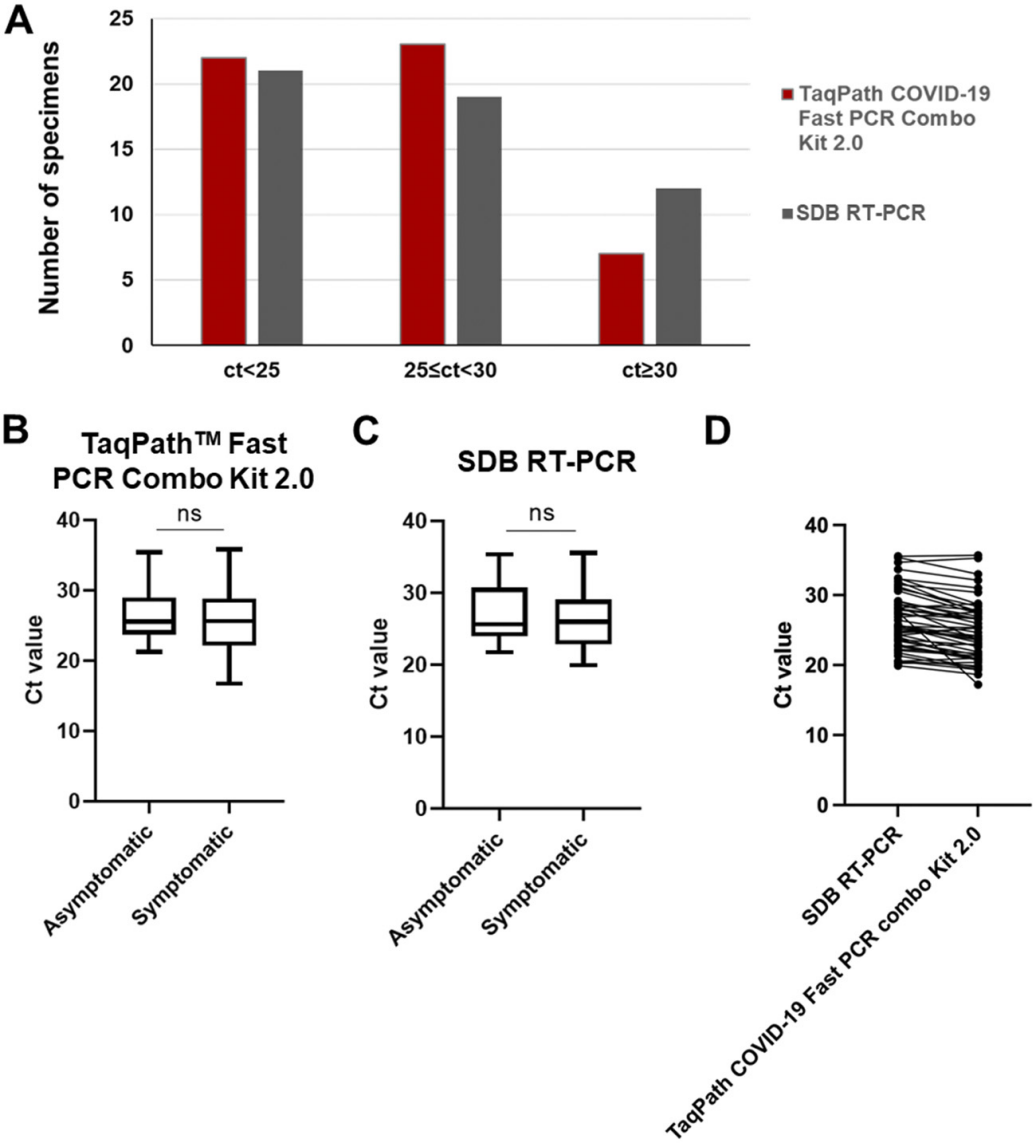
Saliva based SARS-CoV-2 testing Ct values. (A) Distribution of samples across high, medium and low viral loads grouped by Ct value detected with TaqPath Fast 2.0 kit or SDB RT-PCR. (B-C) Comparison of the median Ct values between the symptomatic and asymptomatic individuals positive for SARS-CoV-2 using either the TaqPath COVID-19 Fast 2.0 kit (B) or the SDB RT-PCR test (C). The box plots show the median (bold horizontal line), interquartile range (box), and total range (whiskers) of detected Ct values. (D) Direct comparison of N1 Ct values for each saliva sample obtained using the SDB RT-PCR and the TaqPath COVID-19 Fast 2.0 kit. ns, not significant.

Of the 52 samples in which SARS-CoV-2 presence was detected using the TaqPath COVID-19 Fast PCR Combo kit 2.0, 42.3% (N = 22) of the samples showed a Ct < 25, 44.2% (N = 23) samples were between 25 ≤ Ct < 30 and 13.4% (N = 7) of the samples were of low viral load – with Ct ≥ 30 (Fig. 2A). Similar sample distribution across the 3 Ct ranges could be observed using the lab’s SDB RT-qPCR (Fig. 2A). The comparison of median Ct values in SARS-CoV-2 positive individuals revealed no significant difference between the symptomatic and the asymptomatic patient cohort using both of the RT-qPCR assays used directly on raw saliva samples (Fig. 2B-C). A direct comparison between N1 Ct values obtained per each sample using both direct methods indicate no significant differences among them (Fig. 2D), confirming that both methods are highly comparable in the determination of the presence of SARS-CoV-2 genetic material in raw saliva samples.

## DISCUSSION

From the outset of the SARS-CoV-2 pandemic, both nucleic acid and antigen-based tests were developed and deployed, with a major focus on nasopharyngeal swabs as the biological sample of choice to be tested. However, while RT-PCR-based testing of RNA extracted from NPS samples has been considered the “gold-standard” for SARS-CoV-2 detection (possibly due to regulatory inertia and path-dependency effects), saliva has emerged during the pandemic as a valuable sampling method to improve SARS-CoV-2 detection and workflows (15). Besides the obvious advantage of self-collection associated with lower costs and reduced risks for viral transmission, (8, 10), raw saliva samples can be processed directly through RT-qPCR assays which reduces processing time and removes costs associated with RNA extraction. In addition, saliva (through droplets and aerosols) constitutes a transmission route for SARS-CoV-2 infection and can contain high viral loads of infectious virus as reported by recent studies (7, 11–13). Thus, direct testing for viral detection in saliva can help monitor viral loads across variant surges and assess risk of transmission.

In the current study, we demonstrate that direct RT-qPCR from raw saliva samples using either our in-house developed SDB-PCR assay or a commercially available CE-IVD marked TaqPath Fast kit enables accurate detection of SARS-CoV-2 in both symptomatic and asymptomatic individuals with PPA of >83% and NPA of >99% compared to the “gold-standard” RNA extraction-based RT-qPCR from nasopharyngeal swabs. Despite the long-term storage (~9 months) of raw saliva samples included in the study, the accuracy between saliva and NPS testing was high. This highlights that raw saliva samples can be easily stored for long periods without the need for expensive additives or preservatives.

A number of studies have investigated the use of saliva as a sample method for SARS-CoV-2 detection in comparison to nasopharyngeal swab testing. Although most of such studies compared RNA extraction-based protocols, the findings of such studies were consistent with our results which used direct RT-qPCR on raw saliva. For saliva samples versus NPS, Pasomsub et al. reported a diagnostic sensitivity of 84.3% and specificity of 98.9% (16), while Yokota et al. reported a sensitivity of 92% and specificity of 99.96% for saliva sample versus NPS (17). Other studies investigating direct PCR saliva based testing obtained comparable results with Moreno-Contreras et al. reporting sensitivity of 86.2% (18) and Vogels et al. a positive agreement of 94.1% and a negative agreement of 90.9% for direct PCR from saliva compared to extraction-based NPS testing (19).

Saliva samples are also the direct agents of transmission of SARS-CoV-2, through droplets and aerosols, thereby allowing for direct testing for presence of the biological agent within its transmission vehicle. Depending on variants, individual factors (genotype, age, health etc.) and immunological status (vaccination, prior exposure), SARS-CoV-2 infections can range from asymptomatic to severe symptoms. In this study, using saliva samples we could detect SARS-CoV-2 infections in both symptomatic and asymptomatic individuals, with no significant differences in viral loads (based on Ct values) between both cohorts (Fig. 2).

The COVID-19 pandemic spurred the emergency use authorization in some countries (e.g., USA) of the SalivaDirect protocol for direct detection of SARS-CoV-2 in raw saliva samples (2, 20). Since the advent of the SalivaDirect protocol there have been a number of studies that have compared sensitivity and specificity of RT-qPCR based detection of SARS-CoV-2 across saliva versus nasal swab samples (18, 20–22). A recent systematic review on 16 unique studies with 5922 unique patients suggested that saliva diagnostic accuracy is similar to that of nasopharyngeal swabs, supporting the need to consider saliva as an alternative to NPS (4). Besides, saliva specimens performed similarly to clinician-collected nasopharyngeal swab specimens (21). A study reported 100% positive agreement (38/38 positive specimens) and 99.4% negative agreement (177/178 negative specimens) by using saliva as specimens from symptomatic patients suspected of having COVID-19 (22). Saliva specimens from COVID-19 confirmed patients even provide greater detection sensitivity and consistency due to an approximately 5× higher viral load compared to nasopharyngeal swabs (20).

There have also been studies that have compared different types of diagnostic tests on saliva samples (e.g., nucleic acid versus antigen tests). For instance, Nagura-Ikeda et al., 2020, analyzed seven different molecular diagnostic tests (five RT-PCR tests, one RT-LAMP test and one antigen test), with or without RNA extraction, in self-collected saliva from mostly symptomatic individuals, involving different time points of saliva collection (23). In that study, 103 positive individuals (both symptomatic and asymptomatic) provided saliva samples which were tested by the seven different methods. While in general the direct RT-qPCR methods for the detection of SARS-CoV-2 in saliva had higher sensitivity than RT-LAMP or antigen tests, differences in sensitivity were reported between the direct RT-qPCR kits evaluated (ranging from 78.6% to 81.6%) (23). The two methods evaluated here showed a higher sensitivity (PPA) and specificity (NPA) rates, compared to the reported ones for similar direct RT-qPCR methods (22, 23) (84.74% and 99.62%, respectively, for SDB RT-PCR and 83.05% and 99.44%, respectively, for TaqPath COVID-19 Fast PCR Combo kit 2.0).

Several studies have also evaluated the use of RT-LAMP in saliva samples for SARS-CoV-2 fast detection. One study compared different RT-LAMP testing methods using saliva or NPS as sample and found similar results to the RT-qPCR NPS “gold standard” when using purified/precipitated RNA from each sample type but with significantly reduced sensitivity when the sample was used directly (a reduction from 93% to 65%) (24). With current protocols it would seem that an RNA precipitation step is essential for enhancing performance of RT-LAMP assays, regardless of whether saliva or NPS is used as a sample (24). Similarly, Taki et al. (2021) compared the use of saliva and NPS samples in RT loop-mediated isothermal amplification (LAMP) in 34 viral positive samples (17 NPS and 17 saliva) and 27 negative samples (13 NPS and 14 saliva) (25). Each sample was used for RNA extraction and for a direct test. LAMP remains a good alternative for both types of specimens when RNA extraction is performed with a sensitivity of 97 to 100%. However, when the direct sample was used as a template without an RNA extraction step, a decrease in sensitivity was observed: this pattern was consistent across both sample types but was particularly noticeable for saliva with a loss of sensitivity of 47%, confirming previous observations (23, 24).

A desirable feature of any diagnostic kit is the ability to detect different variants, particularly for the case of RNA viruses such as SARS-CoV-2 that are prone to mutation and recombination. While genome sequencing is ideal for characterization of individual samples, large-scale testing based on genome sequencing has not to date been scaled for everyday practice. All diagnostic tests for SARS-CoV-2 face the challenge of a constantly mutating viral population with periodic emergence of viral variants that display fitness advantages that promote their transmission (26). For nucleic acid based tests, such challenges to detect new variants arise for homology-based molecular tests (e.g., PCR, LAMP) where the mutations (indels) arise in regions that are detected by sequence homology of the diagnostic test (e.g., the primers) (27).

For protein (antigen) based tests, there is potential for reduced affinity and avidity of binding between diagnostic antibodies and antigen polypeptides which can differ between different variants of SARS-CoV-2. Indeed, a lower detection sensitivity (42.8%) during antigen testing has been found for SARS-CoV-2 variants containing SNPs associated with beta or gamma variants (28). In that study, 55 NPSs samples (with different Ct values for RT-qPCR, i.e., different viral loads) were used. The antigen test sensitivity was lower when Ct values were higher (90% when 20 < Ct < 25; 10% when 25 < Ct < 30; 0% when 30 < Ct < 35 and above 35), in comparison with RT-qPCR results (28). Additionally, in the presence of different variants carrying the K417N/T, E484K, and N501Y substitutions, the antigen test sensitivity decreased even when the viral load was high (42%). The underlying reasons for reduction in sensitivity for some antigen tests are unclear but may be due to resulting changes to the tertiary structure of the antigens from the variants in question. Indeed, several studies have found possible effects on antigen test sensitivity with different strains (29, 30). For instance, Bekliz et al., 2022 found variability of effectiveness for detecting the Omicron variant in a study using eight antigen tests (29).

While antigen tests typically cannot distinguish different variants, nucleic acid-based tests which are based on sequence homology can be designed to discriminate between different lineages and variants. The primers used in the Thermo Fisher RT-qPCR kit were designed to help mitigate the risk of unintentional loss of detection of novel variants by amplifying from three different targets on the viral genome, in addition to the positive control for host (human) RNA. The primers for RT-qPCR in the Thermo Fisher kit have also been designed to target regions that are not considered to be hot spots for mutation in the SARS-CoV-2 viral genome, thereby providing some level of futureproofing for detection of both circulating and emerging viral variants. In addition, internal controls are critical for robust identification of positive samples.

In addition to structural differences between variants at the nucleic or polypeptide levels, the viral load and clearance across tissues and disease stages can potentially differ between variants which in turn could have an impact on what biological specimens are most suitable for detecting different variants. Indeed, the Omicron SARS-CoV-2 variant poses a significant challenge for nasal swab based testing as there are indications that saliva based samples may be more effective for diagnostic detection of the omicron SARS-CoV-2 variant relative to NPSs (12, 13).

### Conclusions.

The current COVID-19 pandemic has highlighted the need for diagnostic testing, screening and surveillance methods that are high-throughput and cost-effective. While point-of-care antigen testing has been deployed at scale globally, the reality is that the detection limit of antigen tests remains poorer than PCR-based methods. However, increasing the throughput of PCR-based testing for more accurate detection of SARS-CoV-2 has been constrained by the use of NPS which are costly and cumbersome to collect. In this study, we demonstrated that highly accurate PCR-based testing can be conducted directly on saliva samples, using a Saliva-Direct based test and a novel CE-IVD marked TaqPath COVID-19 Fast PCR Combo kit 2.0. Saliva-based testing for SARS-CoV-2 provides a highly scalable and accurate approach for rapid detection of SARS-CoV-2 especially during surges of COVID-19 cases, for large-scale mass-testing which includes screening and surveillance programs.

## MATERIALS AND METHODS

### Clinical specimens.

Saliva samples from 615 individuals were collected in the Republic of Ireland (Galway) between February and May 2021 at two locations (Airport Testing Centre and National University of Ireland Galway). All individuals provided a signed informed consent, and the study was approved by the NUI Galway Research Ethics Committee (Approval Number: 2020.08.016; Amend 2102). Of the 615 individuals, 39.7% (N = 244) were symptomatic, 35.3% (N = 217) were asymptomatic, while the information on the symptomatic status was not available for the remaining part of the cohort (Fig. 1A). The Asymptomatic or Symptomatic status of each individual was assigned based on answer given to the question “*Reason why you are being tested by the HSE”* in the registration form that was provided to each volunteer. Each individual who indicated that they had a cough and/or high temperature were classified as symptomatic, while others were classified as asymptomatic (such individuals had been referred for testing as they had been contact traced in accordance with the government guidelines of the time). All saliva samples were tested upon collection using the SalivaDirect-based RT-PCR. Concurrent to saliva collection, NPS were collected and tested for SARS-CoV-2 presence using an RNA extraction-based method according to the national COVID-19 testing system in Ireland run by the Health Service Executive (HSE). Following circa 9 months of storage at −20°C, raw saliva samples were thawed and retested using the lab’s SDB RT-qPCR as well as the TaqPath COVID-19 Fast PCR Combo kit 2.0. Exclusion criteria included: inconclusive result on the TaqPath COVID-19 Fast PCR Combo kit 2.0 and altered status prior to and following storage on the SDB RT-PCR test.

### SARS-CoV-2 detection.

Raw saliva samples were tested upon collection and following storage using the SDB RT-PCR test. In brief, the samples were treated according to the SalivaDirect protocol; 25 μL of each raw saliva sample was collected on a 2.0 mL Eppendorf tube and treated with Proteinase K at 2.5 μg/μL final concentration followed by heat inactivation at 95°C for 5 min. RT-PCR was performed on the Applied Biosystems StepOnePlus real-time PCR system using the Applied Biosystem TaqMan Fast Virus 1-Step Master Mix together with the CDC2019-Novel Coronavirus Real-time RT-PCR diagnostic panel and results analyzed using the StepOne Software v2.3. In parallel, saliva samples were tested using the TaqPath COVID-19 Fast PCR Combo kit 2.0 according to the manufacturer’s instructions. Briefly, 20 μL of the SalivaReady solution were added to 20 μL of each raw saliva samples and mixed. 20 μL of each mix was transferred to a 96-well plate, sealed and vortexed followed by 5 min incubation at 62°C and 5 min incubation at 92°C. Plates were maintained on ice while preparing the RT-qPCR. 14 μL of the treated saliva samples were used. The TaqPath COVID-19 Fast PCR Combo kit 2.0 is a fast direct PCR, without RNA extraction, which includes 8 targets across 3 SARS-CoV-2 genes (Orf1a, Orf1b and N) to ensure accurate detection of SARS-CoV-2 as new mutations continue to arise. RT-qPCR was performed on the QuantStudio 5 Real Time PCR Instrument with QuantStudio Design and Analysis software v1.5.1, and results were analyzed using the Pathogen Interpretive Software CE-IVD Edition 1.1.0. NPS samples were tested within the national HSE testing program using an RNA extraction-based RT-PCR with the TaqPath COVID-19 CE-IVD RT-PCR kit. The study design is shown in Fig. 1B.

### Whole-genome sequencing of SARS-CoV-2.

Whole-genome sequencing (WGS) of a subset of the SARS-CoV-2 positive saliva samples (N = 46) was performed on RNA extracted from saliva samples via a Quick DNA/RNA Viral MagBead kit (Zymo, R2140). RNA samples were sent on dry ice to the Quadram Institute Bioscience, UK for WGS of SARS-CoV-2. Viral RNA was converted in cDNA and then amplified using the ARTIC protocol v3 (LoCost) (31) with sequencing libraries prepared using CoronaHiT (32). WGS was performed using the Illumina NextSeq 500 platform with one positive control and one negative control. The raw reads were demultiplexed using bcl2fastq (v2.20). The reads were used to generate a consensus sequence using the ARTIC bioinformatic pipeline (https://github.com/connor-lab/ncov2019-artic-nf). Briefly, the reads had adapters trimmed with TrimGalore (33), and were aligned to the Wuhan Hu-1 reference genome (accession MN908947.3) using BWA-MEM (v0.7.17) (34); the ARTIC amplicons were trimmed and a consensus built using iVAR (v.1.3.0) (35). Genomes that contained more than 10% missing data were excluded from further analysis to ensure high quality phylogenetic analysis. PANGO lineages were assigned using Pangolin (v2.3.2) (https://github.com/cov-lineages/pangolin) and PangoLEARN model dated 2021-02-21 (36).
